# Anterior Capsular Tear During Phacoemulsification: Successful Management With the Two-Y Crushing Technique

**DOI:** 10.7759/cureus.104748

**Published:** 2026-03-05

**Authors:** R Balamurugan, Jyothi K Velivela, Shubhangi SN Prasad, Kiratmeet Singh, Shagila R

**Affiliations:** 1 Ophthalmology, All India Institute of Medical Sciences, Madurai, Madurai, IND; 2 Ophthalmology, All India Institute of Medical Sciences, Mangalagiri, Mangalagiri, IND

**Keywords:** anterior capsular tear, cataract surgery, errant capsulorhexis, intraoperative complication, phacoemulsification, runaway capsulorhexis, two-y crushing technique

## Abstract

An anterior capsular tear or radial extension of the capsulorhexis is a serious complication during phacoemulsification and may lead to significant sequelae. In the present case, an anterior capsular tear occurred during capsulorhexis in phacoemulsification surgery. We opted to use the Two-Y crushing technique to safely emulsify and aspirate the nucleus in the presence of an anterior capsular tear. This technique was originally described for nuclear cracking in posterior polar cataracts (PPCs) under the protection of an epinuclear shell, with the advantage of minimizing capsular stress. In this technique, following successful hydrodelineation and viscodelineation, the nucleus was prolapsed out of the capsular bag, crushed into multiple fragments using two Y-rotators, and subsequently emulsified using a phaco probe in the anterior chamber at the iris plane. Throughout the procedure, minimal stress was exerted on the torn anterior capsule, and with appropriate precautions, the surgery was completed successfully with a favorable visual outcome.

## Introduction

Continuous curvilinear capsulorhexis (CCC) is a crucial step in phacoemulsification cataract surgery, as it enables safe emulsification and removal of the lens within the capsular bag using a phaco needle (endocapsular phacoemulsification). However, during the performance of CCC, particularly among beginners, and occasionally even in experienced hands, or in the presence of risk factors such as positive vitreous pressure, an intumescent cataract with high intralenticular pressure, or a shallow anterior chamber, there is an increased tendency for radial extension or tear-out of the anterior capsule [1]. A radial tear of the anterior capsule may further extend to the equator, zonules, and posterior capsule, resulting in posterior capsular rupture (PCR), nucleus drop, vitreous loss, and inadequate capsular support for intraocular lens (IOL) implantation [1]. Several techniques have been described to manage anterior capsular tears, including the midway tangential anterior capsular flap technique [1], the Little rescue technique [2], the quick-pull technique [3], anterior zonulotomy [4], safety capsulorhexis [5], anterior chamber phacoemulsification [6], rhexis redirection from the opposite side [2,4,5,7] or from the margin of the rhexis flap [2,4,5,7], and the slow-motion phacoemulsification technique [8]. During endocapsular phacoemulsification, if capsular compromise occurs, such as an anterior capsular tear, posterior capsular tear, or a preexisting dehiscent posterior capsule, the primary goal is to debulk the nucleus as quickly and safely as possible to minimize the risk of nucleus drop. Nucleus debulking with minimal or no stress on the capsule can be easily performed using the two-Y crushing technique after successful hydrodelineation [9]. The Two-Y crushing technique [9] is a simple method originally introduced for the management of the nucleus in posterior polar cataract (PPC). In this technique, the endonucleus is prolapsed into the anterior chamber using a Y-rotator after hydrodelineation and viscodelineation, without performing hydrodissection, and then crushed into multiple fragments (at least four pieces) in the anterior chamber. Subsequent phacoemulsification is performed under the protection of dispersive viscoelastic (hydroxypropyl methylcellulose (HPMC) 2%) with a protective epinucleus shell. This approach reduces ultrasonic energy usage, facilitates easy nucleus debulking in the anterior chamber, and minimizes stress and manipulation on the dehiscent or compromised capsular bag. In the present case, we successfully adapted the Two-Y crushing technique to debulk the nucleus in an inadvertent radial extension of the anterior capsulorhexis encountered during CCC in phacoemulsification cataract surgery. In the setting of an anterior capsular tear, our primary aim was to achieve maximum nuclear debulking while minimizing capsular stress, thereby preventing further extension of the capsular tear. This was safely and effectively accomplished using the Two-Y crushing technique.

## Case presentation

**Figure 1:**
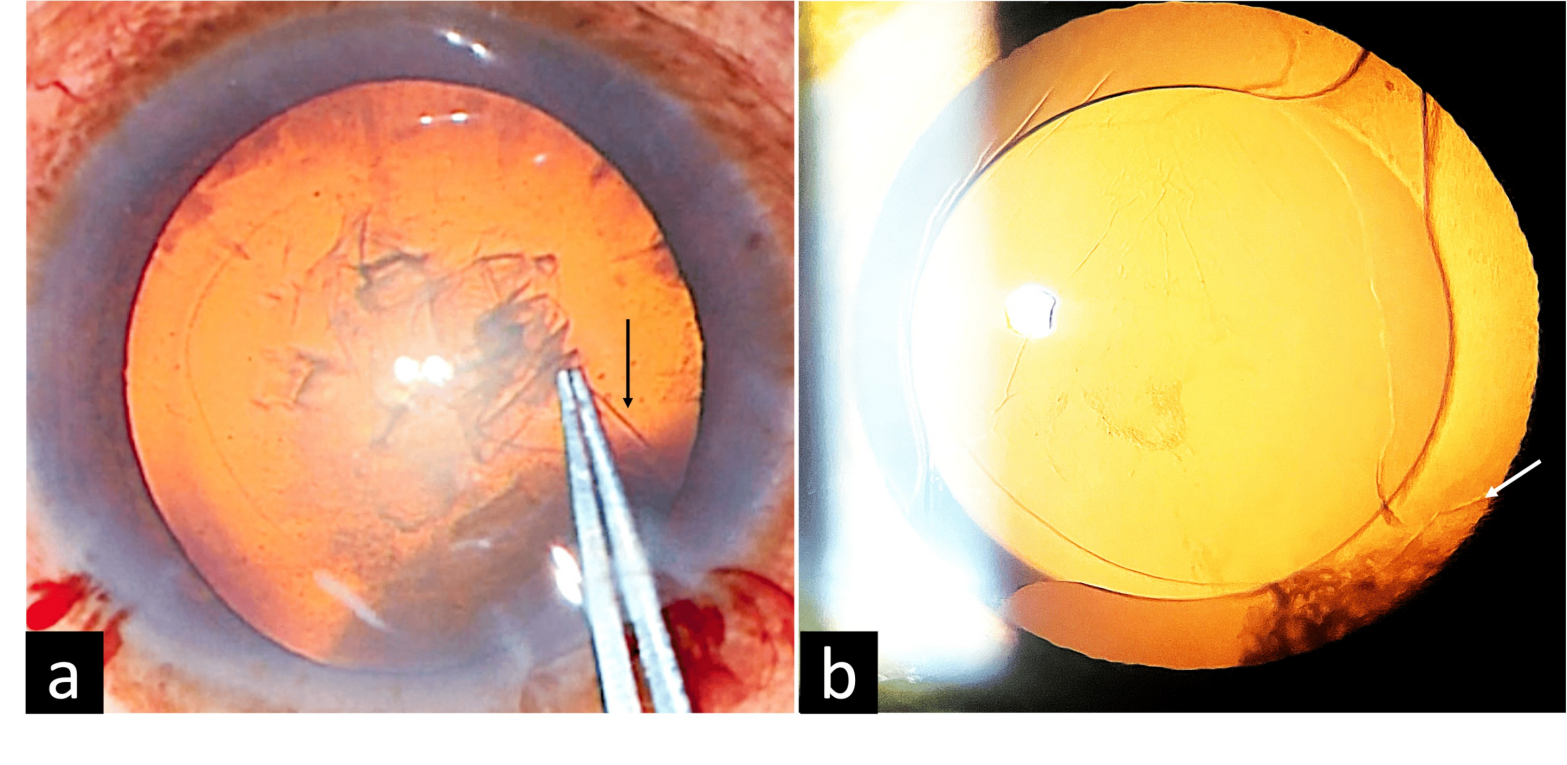
Pane 1a demonstrates the radial pull of the extended or runaway capsule (black arrow) on attempting capsulorhexis using Utrata rhexis forceps. Pane 1b shows a well-centered single-piece intraocular lens in the bag, with both haptics well placed under adequate anterior capsular support.

**Figure 2:**
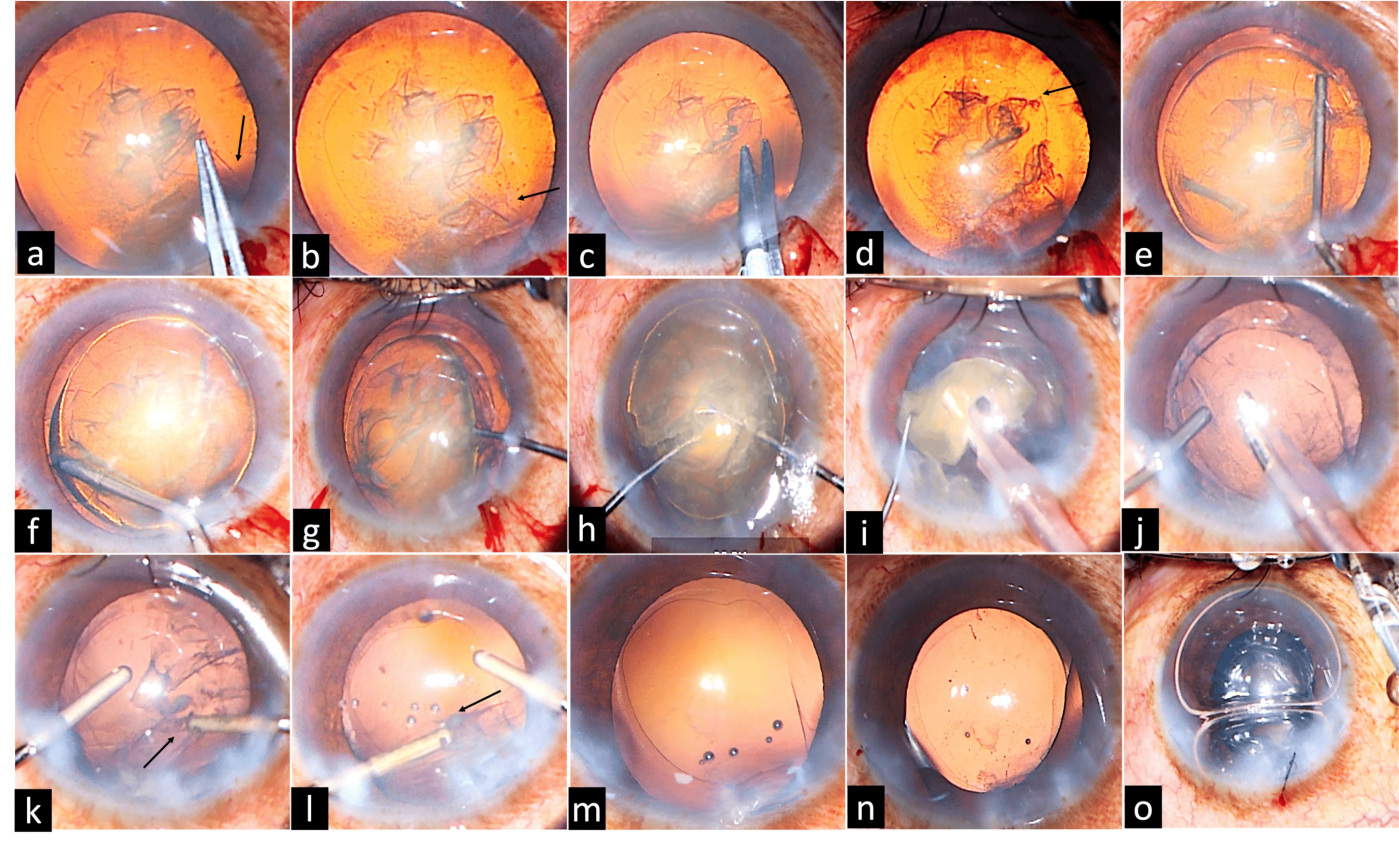
Pane 2a shows the radial pull of the extended or runaway anterior capsule (black arrow) on attempting capsulorhexis using Utrata capsulorhexis forceps; Pane 2b shows the extended anterior capsule; Pane 2c shows a nick given at the margin of the anterior capsule; Pane 2d shows completion of the capsulorhexis; Pane 2e shows hydrodelineation performed to separate the nucleus from the epinucleus; hydrodissection was avoided to prevent further extension of the capsular tear; Pane 2f shows viscodelineation performed to adequately separate the endonucleus; Pane 2g shows the endonucleus prolapsed into the anterior chamber using a Y-rotator. Pane 2h shows the nucleus crushed using two Y-rotators into at atleast four pieces (Two-Y crushing technique); Pane 2i shows removal of the nucleus using phacoemulsification; Pane 2j shows injection of viscoelastic (hydroxypropyl methylcellulose 2%) before removing the phaco probe or any irrigation source to prevent collapse of the anterior chamber; Panes 2k and 2l show cortical removal was carried out using an irrigation and aspiration probe, and near the area of the capsulorhexis radial tear-out, the direction of cortex removal should be toward the tear; Pane 2m shows clear visualization of the radial tear extending from the anterior capsule after removal of all lens material; Pane 2n shows a well-placed intraocular lens in the capsular bag with the haptics positioned under adequate anterior capsular support; and Pane 2o shows the anterior chamber formed with an air bubble, with the main port sutured in anticipation of the planned intravitreal injection.

A 54-year-old male patient with diabetes presented with grade III nuclear sclerosis cataract and diabetic macular edema (DME) associated with mild non-proliferative diabetic retinopathy (NPDR) in the left eye, while the right eye had grade I nuclear sclerosis without diabetic retinopathy. Best-corrected visual acuity (BCVA) was 6/9 (−1.00 diopter sphere (DS)/−0.75 diopter sphere (DC) × 90) in the right eye and 6/24 (−2.50 DS) in the left eye. Phacoemulsification with IOL implantation, along with intravitreal anti-vascular endothelial growth factor (anti-VEGF) injection in the left eye, was undertaken (Video 1). During continuous CCC in the left eye, the rhexis inadvertently extended into the periphery, resulting in an anterior capsular tear (runaway, extended, or tear-out capsulorhexis), as shown in Figures 1a, 2a, 2b. A nick was made at the rhexis margin (Figure 2c) to complete the rhexis (Figure 2d) [2]. Hydrodissection was avoided to prevent further extension of the tear [6], and hydrodelineation was performed to separate the endonucleus from the epinucleus (Figure 2e), followed by viscodissection using HPMC 2% to achieve adequate nuclear separation (Figure 2f). A Y-rotator was used to prolapse the nucleus into the anterior chamber (Figure 2g) [9], and the nucleus was subsequently crushed into a minimum of four fragments using the Two-Y crushing technique (Figure 2h), after which phacoemulsification of the nucleus was completed at the iris plane under the protection of adequate dispersive viscoelastic (HPMC 2%) with a protective epinucleus sheet underneath without giving direct stress to the capsule (Figure 2i). Prior to withdrawing the phaco probe or any irrigation source, dispersive viscoelastic (HPMC 2%) was injected to prevent anterior chamber collapse and further extension of the capsular tear (Figure 2j) [5]. The epinucleus separated spontaneously from the cortical plane; otherwise, it should be safely separated using viscodissection with HPMC 2%. Cortical aspiration was performed using an irrigation-aspiration (I and A) probe, with particular care taken to remove the cortex adjacent to the capsular extension by directing traction toward the tear (Figures 2k, 2l) [5]. Aspirating or dragging the cortex away from the tear can lead to further extension of the rhexis and should be avoided. Following complete removal of lens matter, the capsular bag was carefully assessed and found to provide adequate support despite the visible radial tear beyond the iris margin (Figure 2m). A single-piece IOL was implanted in the bag, with both haptics positioned securely beneath the region of an intact and adequate anterior capsular rim (Figure 2n) [5]. The anterior chamber was well formed with an air bubble, and the main port was sutured (Figure 2o) in view of the planned intravitreal anti-VEGF injection, which was subsequently administered [10]. At the end of surgery, the IOL was well centered with a stable anterior chamber, and at three months’ follow-up, the patient achieved a BCVA of 6/6 (−1.25 × 100) with a well-centered IOL (Figure 1b) and complete resolution of DME.

**Video 1:** This video demonstrates the surgical steps for managing an anterior capsular tear using the Two-Y crushing technique

## Discussion

If an anterior capsular tear occurs during phacoemulsification, the primary objective of the surgeon should be to debulk the nucleus while exerting minimal stress on the margin of the tear [6]. Various techniques have been published describing the management in the current situation, either to rescue the tear or rhexis with endocapsular phacoemulsification or anterior chamber phacoemulsification. Techniques that allow rescue of the capsular flap followed by endocapsular phacoemulsification include the midway tangential anterior capsular flap technique [1], in which a midway tangential anterior capsular flap is created, grasped with forceps, and pulled toward the center, allowing the flap to rejoin the edges of the extended rhexis. In the little rescue technique [2], the capsular flap is unfolded, repositioned to its original anterior capsular location, and then pulled in the opposite direction. In the quick-pull technique [3], the extended flap is rapidly pulled centrally in the plane of the anterior capsule. In anterior zonulotomy [4], when a rhexis tear extends into the zonules, the zonules along the intended redirection pathway are cut, thereby facilitating the application of other capsular flap rescue techniques. The Two-Y crushing technique [9] offers several advantages in this situation, as it facilitates safe removal of the nucleus in the anterior chamber at the iris plane under the protection of dispersive viscoelastic with a protective epinucleus sheet, thereby minimizing stress on the capsular bag and preventing further extension of the tear. The manual fragmentation of the nucleus using the Two-Y rotator may decrease the utilization of ultrasonic phaco energy, as only 2.4 cumulative dissipated energy (CDE) was used to completely emulsify the grade III hard nucleus. The key precautions while performing the Two-Y crushing technique in the presence of an anterior capsular tear include achieving successful hydrodelineation followed by viscodelineation to separate the endonucleus from the epinucleus; avoiding hydrodissection [6]; injecting adequate dispersive viscoelastic to protect the corneal endothelium from phaco energy; performing phacoemulsification at the iris plane; using viscodissection to separate the epinucleus from the cortex; and directing cortical removal toward the capsular tear [5], as dragging or aspirating the cortex away from the tear can lead to further extension and should be avoided. In-the-bag IOL implantation should be performed with both haptics securely positioned beneath the region of the intact anterior capsular rim [5]; however, sulcus placement of a three-piece IOL may be considered in cases of inadequate anterior capsular support or PCR with adequate sulcus support [5]. This may be followed by a pilocarpine injection to stabilize the IOL-bag complex and the formation of the anterior chamber with an air bubble. Phacoemulsification settings should ideally be kept low, with ultrasound energy around 50%-60%, bottle height 55-70 cm, vacuum 30-100 mmHg, and an aspiration flow rate of 15-25 mL/min [11]. This technique should not be attempted in cases of failed hydrodelineation and may be challenging in eyes with a poorly dilating pupil or a shallow anterior chamber.

## Conclusions

An anterior capsular rhexis tear or runaway rhexis is a serious intraoperative complication that can lead to significant sequelae if not managed appropriately. Although multiple management strategies exist, their application depends on the surgeon’s experience and intraoperative judgment. The Two-Y crushing technique, originally established as a safe method for nuclear debulking in PPCs with minimal capsular stress, can be safely adopted for the management of anterior capsular rhexis tears with appropriate precautions. Further studies and larger case series are required to establish its effectiveness.
